# The Ca^2+^-Regulated Protein Kinase CIPK1 Modulates Plant Response to Nitrate Deficiency in *Arabidopsis*

**DOI:** 10.3390/genes15091235

**Published:** 2024-09-23

**Authors:** Hang Su, Qian Wang, Lihu Wang, Junjun Cui

**Affiliations:** 1 School of Landscape and Ecological Engineering, Hebei University of Engineering, Handan 056038, China; wanglihu@hebeu.edu.cn (L.W.); cuijunjun@hebeu.edu.cn (J.C.); 2 Research Center for Stress Physiology in Fruit Trees, Hebei University of Engineering, Handan 056038, China; 3 Library, Hebei University of Engineering, Handan 056038, China; wqhbgc2023@126.com

**Keywords:** *Arabidopsis thaliana*, nitrate deficiency, Ca^2+^ signaling, CIPK1

## Abstract

Background/Objectives: Nitrogen is an essential macroelement for plant growth and productivity. Calcium (Ca^2+^) acts as a critical second messenger in numerous adaptations and developmental processes in plants. The Calcineurin B-like protein (CBL)-interacting protein kinase (CIPK) signaling pathway has been demonstrated to be involved in multiple intracellular ion homeostasis of plants in response to stresses. However, whether CIPKs are involved in nitrate deficiency stress remains largely unknown. Methods: In this study, we screened *Arabidopsis thaliana* T-DNA insertion mutants of the CIPK family under nitrate deficiency conditions by a reverse genetic strategy. Results: We found that the *cipk1* mutant showed a shorter primary root and had a lower fresh weight and total N content compared with wildtype (WT) plants under nitrate deficiency. The *CIPK1* complementation lines completely rescued the sensitive phenotype. Additionally, *CIPK1* mutation caused nitrogen-starvation marker genes to be decreased under nitrate deficiency. We further found that CIPK1 interacted with teosintebranched 1/cycloidea/proliferating cell factor 1-20 (TCP20) in a yeast two-hybrid system. Conclusions: Collectively, our results reveal a novel role of CIPK1 in response to nitrate deficiency in *Arabidopsis*.

## 1. Introduction

Nitrogen (N) is an essential mineral element required in the largest amounts by plants, and its availability is an important factor for the normal growth and development of plants [1,2]. N nutrients can be classified as inorganic forms like nitrate and ammonium, and organic forms such as amino acids and urea. Nitrate is the major source of N in agricultural and many other environments [3]. However, the nitrate supply in natural or agro-ecosystems soils is often insufficient. The application of N fertilizer has increased remarkably in recent years to obtain high yields. N fertilizer consumption has reached almost 120 million tons per year worldwide, accounting for 59% of total fertilizer nutrient (FAO, 2017). However, crops can only use roughly 30 to 50% of the N applied from fertilizers [4]. The massive supply of inorganic N fertilizers has contributed to enable maximal crop output over the past century. However, the overuse of fertilizers has also led to environmental pollution, such as the eutrophication of land and aquatic systems. Therefore, reducing excess N application to soil while maintaining high crop yields is a global goal for breeders. A better understanding of the nitrate absorption mechanism and nitrogen use efficiency (NUE) in plants is key for achieving this goal [5].

Nitrate is the major nitrogen source for most land plants, and besides acting as a nitrogen source, nitrate has been demonstrated to serve as a signal molecule that modulates plenty of plant physiological processes, such as seed germination, root architecture, shoot development, circadian rhythms, and flowering [6,7,8,9,10]. Plants actively acquire nitrate from fluctuating environments by a proton/nitrate-coupled machinery [11]. The identification of genes encoding nitrate uptake carriers has found 53 *nitrate transporter1* (*NRT1*)/*peptide transporter* (*PTR*) (*NPF*) genes and 7 *NRT2* genes in *Arabidopsis* [12,13,14,15]. Among them, most NPF transporters display low nitrate affinities, while NRT1.1/NPF6.3/chlorate resistant 1 (CHL1) displays dual affinities [16]. In general, NRT2s likely display high nitrate affinities. Nitrate regulates a transcriptional response called the primary nitrate response (PNR), which begins with the perception of external nitrate by NRT1.1 [12,16,17,18]. Since nitrate is sensed by NRT1.1, the signal needs to be communicated to the nucleus to manage gene expression. Many transcription factors involved in the nitrate response have been identified, such as NIN-like protein 6/7 (NLP6/7), TGACG motif-binding factor 1/4 (TGA1/4), and teosintebranched 1/cycloidea/proliferating cell factor 1–20 (TCP20) [19,20,21,22]. Nevertheless, the molecular mechanisms governing nitrate acquisition and signaling remain predominantly ambiguous.

In plants, calcium (Ca^2+^) is a conserved and versatile signaling modulator in response to biotic and abiotic stress, immunity, nodulation, and circadian rhythms [23]. The Ca^2+^ signatures are decoded by sensors such as Ca^2+^ binding proteins. The calcineurin B-like (CBL) protein family act as a group of Ca^2+^ sensors, specifically interacting with, and regulating a family of unique protein kinases in plants, defined as CBL-interacting protein kinases (CIPKs) [24]. The CIPK network has been demonstrated to be involved in multiple intracellular ion homeostasis. The CBL1/9-CIPK23 complex can positively activate *Arabidopsis* K^+^ transporter 1 (AKT1) and high-affinity K^+^ transporter 5 (HAK5) in response to K^+^ deficiency, and phosphorylate NRT1.1 at Thr101 to affect nitrate uptake and signaling [25,26,27,28]. The CBL4-CIPK24 complex regulates plasma membrane Na^+^/H^+^ antiporter in the salt overly sensitive (SOS) pathway [29,30]. CBL2/3-CIPK3/9/23/26 complexes have been demonstrated to regulate vacuolar Mg^2+^ sequestration in response to Mg^2+^ toxicity stress [31]. The Ca^2+^-dependent phosphorylation of manganese, cadmium, arsenate, and iron transporters by CBL-CIPKs and CPKs has been found [32,33,34,35,36,37,38]. Furthermore, CIPK8 has been found as a positive regulator in low-affinity nitrate response [39]. CBL7 has been found to be involved in the regulation of nitrate deficiency response [40]. The phosphorylation of CIPK15 mediates the feedback inhibition of ammonium transporter 1 (AMT1) [41]. CPK6 has been found to phosphorylate NRT1.1, repressing the transporting activity under high N and drought stress [42]. CIPK1 phosphorylated NAC transcription factor NAC075, leading to the transcriptional regulation of downstream target WRKY53, consequently leading to adapted root architecture under low-nitrate availability. However, no significant difference was observed in primary root growth between the wildtype (WT) and the *cipk1* mutants. However, the overexpression of *CIPK1* led to decreased sensitivity to low nitrate in terms of root elongation [10]. Whether there exist other roles of CBL-CIPKs in modulating nitrate uptake and signaling is still unclear.

In this study, we report that CIPK1 acts as a positive regulator in response to nitrate deficiency. The *cipk1* mutant exhibited a more sensitive phenotype under nitrate starvation than WT. The mutation of *CIPK1* decreases total N accumulation in *Arabidopsis* and represses N-starvation marker genes under nitrate deficiency, such as *NRT1.1* and *NRT2.1*. Using a yeast two-hybrid system, we further found that CIPK1 interacted with transcription factor TCP20. Taken together, our findings provide new insight into CIPK1 in response to nitrate deficiency.

## 2. Materials and Methods

### 2.1. Plant Material and Growth Conditions

*Arabidopsis* in the Wassilewskija (Ws) background was employed as WT. The *cipk1* T-DNA insertion mutant (N9889) and complementation lines were obtained from our previous study [43]. For phenotypic assays, seeds were surface-sterilized at 5% (*v*/*v*) NaClO and 0.05% (*v*/*v*) Triton X-100, and then kept in darkness at 4 °C for 2 days until stratification. Seeds then were sown on 1/2 MS medium (Sigma, Kanagawa, Japan), supplemented with 1% (*w*/*v*) sucrose and 1% (*w*/*v*) agar (Solarbio, Beijing, China), pH 5.8, and vertically placed in a growth cabinet under a 22 °C/19 °C and 16 h/8 h light/dark regime. Uniform 4-day-old seedlings of characteristic size for each genotype were transferred to N-deficient MS medium (MSP07, Caisson), supplemented with 0, 0.01, and 0.05 mM KNO_3_, sucrose, and agar as described above. N-deficient MS medium supplemented with 20 mM NO_3_^−^ was used as the NO_3_^−^-sufficient control group, which has the same NO_3_^−^ concentration with regular 1/2 MS medium. The primary root length and lateral root density treated on different nitrate concentrations were computed after 7 days using ImageJ software (1.38e).

For germination assays, seeds were surface-sterilized at 5% (*v*/*v*) NaClO and 0.05% (*v*/*v*) Triton X-100, and then kept in darkness at 4 °C for 2 days until stratification. Seeds then were sown on NO_3_^−^-sufficient control group medium or N-deficient MS medium, supplemented with 0, 0.05, 0.1, and 1 mM KNO_3_, sucrose, and agar as described above. After 10 days, the primary root length grown on diverse nitrate concentrations were calculated using ImageJ software.

### 2.2. Determination of Total N Content

To analyze the fresh weight and total N content, 7-day-old seedlings germinated on 1/2 MS medium were transferred to the modified Hoagland solution (1 mM KCl, 1 mM CaCl_2_, 0.4 mM MgSO_4_, 0.2 mM KH_2_PO_4_, 3 μM H_3_BO_3_, 1 μM Na_2_MoO_4_, 0.4 μM ZnSO_4_, 0.2 μM CuSO_4_, and 20 μM FeNa-EDTA) for a week and then transferred to the control Hoagland solution or N deficient (LN 0.05 mM) Hoagland solution for another week under a 22 °C/19 °C and 16 h/8 h light/dark regime. The shoots and roots of plants were separately harvested and dried at 65 °C for 3 days. The samples were boiled through a H_2_SO_4_-H_2_O_2_ method for nitrogen analysis using a high-resolution automatic chemical analyzer (SEAL, AA3).

### 2.3. Gene Expression Analysis

For gene expression analysis, seedings were grown on 1/2 MS medium for 7 days, and then transferred to control medium or nitrate-deficient medium for 2 days. Total RNA was extracted using the TRIZOL reagent kit (TIANGEN, Beijing, China). The first-strand cDNA was synthesized suing the FastKing RT Kit with gDNase (TIANGEN). Quantitative real-time PCR was implemented using ChamQ SYBR qPCR Master Mix (Vazyme, Nanjing, China) on a CFX96 connect system (Biorad, Hercules, CA, USA). The *ACTIN2* gene was employed as an internal control. The primers used are listed in Table S1.

### 2.4. Yeast Two-Hybrid Assay

For the interaction of CIPK1 with potential candidates, full-length cDNAs of interest were cloned. The method was previously described [44].

### 2.5. Statistical Analysis

Data are given as means ± standard error (SE) of one representative experiment with *n* ≥ 4 individual plants. Each experiment was repeated independently at least two times with similar results. The significance between the means of different treatments/genotypes was evaluated by Student’s *t* tests using SPSS version 19.0 software (IBM, Armonk, NY, USA).

## 3. Results

### 3.1. The cipk1 Mutant Shows Growth Inhibition Phenotype to Nitrate Deficiency

**Figure 1:**
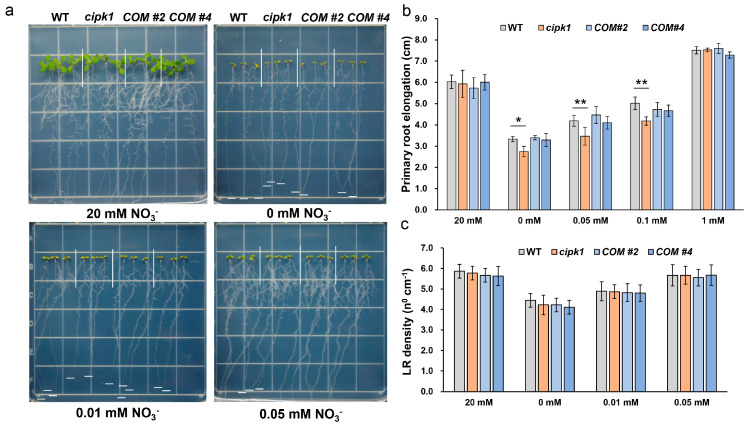
The *cipk1* mutant is more sensitive to nitrate deficiency. (**a**) Phenotypic analysis of the *cipk1* mutant and complementation lines under 0, 0.01, 0.05, and 20 mM nitrate. (**b**) The primary root lengths of the evaluated lines under various nitrate treatment conditions (*n* = 24). (**c**) Lateral root density under various concentrations of nitrate treatments (*n* = 24). Error bars indicate means ± S.E. Asterisks denote statistically significant differences according to the *t*-test (* *p* < 0.05, ** *p* < 0.01).

To determine whether members of the CIPK family of proteins are involved in N deficiency responses in *Arabidopsis*, we performed a reverse genetic approach, screening CIPKs T-DNA insertion mutants in N deficiency (Figure S1). As N deficiency stimulates the elongation of primary roots and the emergence of lateral roots, we used four-day-old uniform seedlings germinated from the control group Murashige and Skoog (MS) medium (20 mM nitrate) and then transferred them to medium containing 0, 0.01, or 0.05 mM KNO_3_ for 7 days. Strikingly, we found that *cipk1* seedlings exhibited growth inhibition phenotype with short primary roots compared with WT under N deficiency while there were no differences in lateral root densities. When grown on the control group medium, no difference was observed between *cipk1* and WT (Figure 1). To validate that the nitrate deficiency-inhibition phenotype of the *cipk1* mutant was caused by *CIPK1* gene loss, *CIPK1* complementation lines served as control. Phenotypic assays showed that the primary roots of transgenic lines #2 and #4 were homogeneous to those of WT and longer than the *cipk1* mutant under N deficiency. These results demonstrate that the nitrate deficiency-inhibition phenotype of the *cipk1* mutant was a consequence of the loss of the *CIPK1* gene.

**Figure 2:**
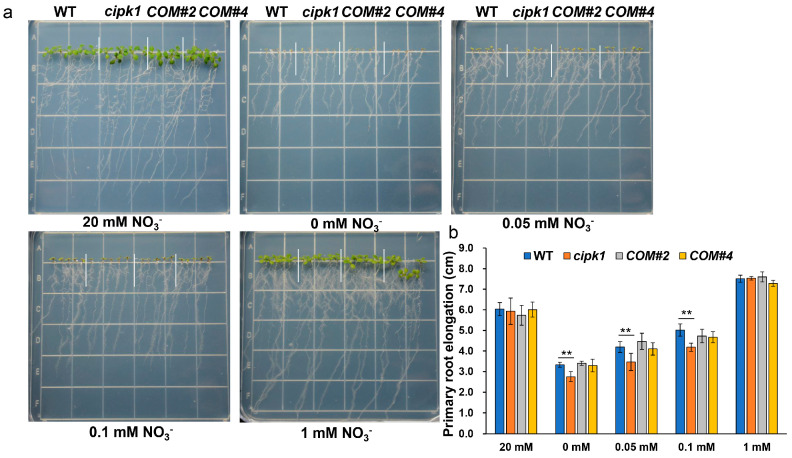
Characterization of the *cipk1* mutant phenotype germinated on LN medium. (**a**) Phenotypic analysis of the *cipk1* mutant and complementation lines germinated from 0, 0.05, 0.1, 1, and 20 mM nitrate. (**b**) The primary root lengths of the evaluated lines under various nitrate treatment conditions (*n* = 24). Error bars indicate means ± S.E. Asterisks denote statistically significant differences according to the *t*-test (** *p* < 0.01).

We then conducted germination experiments to further verify phenotypic differences in the *cipk1* mutant in N deficiency, using seeds germinated and grown on control group and medium containing 0, 0.05, 0.1, or 1 mM KNO_3_ for 7 days. Like the results above, the *cipk1* mutant also displayed a sensitive phenotype with shorter primary roots compared with WT under N deficiency (Figure 2), while the transgenic lines #2 and #4 were homogeneous to WT. In conclusion, these results indicate that CIPK1 plays a positive role in response to N deficiency in *Arabidopsis*.

### 3.2. Mutation of CIPK1 Decreases Total N Accumulation

**Figure 3:**
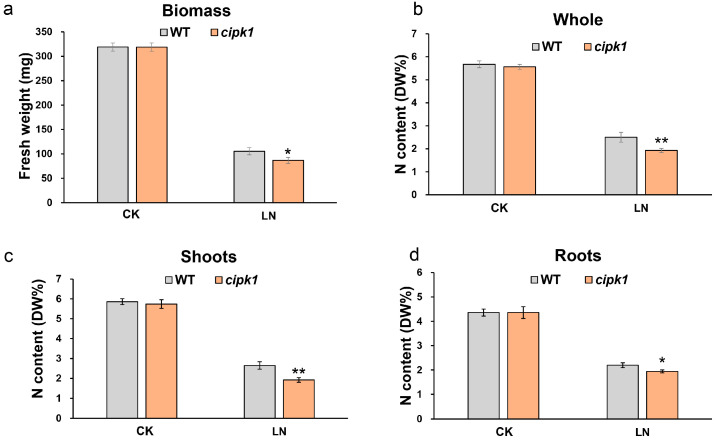
Loss of *CIPK1* decreases N accumulation. (**a**) Biomass analysis of *cipk1* mutant under 20 mM nitrate (CK) and nitrate deficiency (LN). (**b**) Total N content in whole plant of *cipk1* mutant under nitrate deficiency. (**c**) Total N content in shoots of *cipk1* mutant under nitrate deficiency. (**d**) Total N content in roots of *cipk1* mutant under nitrate deficiency. (*n* = 24). Error bars indicate means ± S.E. Asterisks denote statistically significant differences according to *t*-test (* *p* < 0.05, ** *p* < 0.01).

CIPK1 has been reported as localized in plasma membrane, cytosol, and nucleus, indicating that it functions in numerous cellular processes. To investigate whether the inhibition phenotype of the *cipk1* mutant under N deficiency was a result of the decreased N accumulation, we examined the fresh weight and total N content of the *cipk1* mutant under N deficiency. Seven-day-old homogenous seedlings germinated on 1/2 MS medium were transferred to modified Hoagland solution for a week and then transferred to control Hoagland solution or N-deficient (LN 0.05 mM) Hoagland solution for another week. We found that the fresh weight of the *cipk1* mutant was lower than that of WT under N deficiency, while there was no difference in control condition (Figure 3). The total N content of the shoots, roots, and whole plant was measured. We found that the shoots, roots, and whole plants accumulated less N content under LN condition compared with WT. No difference was observed under the control condition. These results indicate that the mutation of *CIPK1* decreases total N accumulation in *Arabidopsis*.

### 3.3. N-Starvation Marker Genes Expression Were Repressed in cipk1 Mutant under N Deficiency

According to the results above, we speculated that *CIPK1* is a positive regulator in response to N deficiency in *Arabidopsis*. We therefore tested whether *CIPK1* may be regulated at the transcriptional level. Then, we performed real-time qRT-PCR using the shoots and roots of seedlings under control or N deficiency conditions. The consequences showed that *CIPK1* mRNA levels had no difference between WT seedlings under control or N deficiency conditions in either shoots or roots (Figure S2).

**Figure 4:**
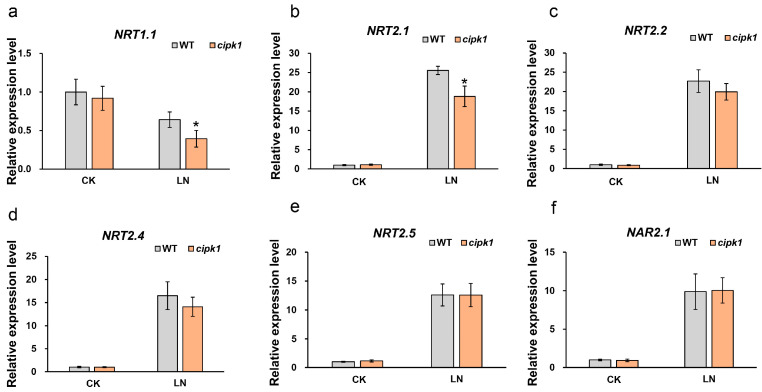
The effect of *CIPK1* mutation on the expression level of N-starvation marker genes. (**a**–**f**) Expression of *NRT1.1*, *NRT2.1*, *NRT2.2*, *NRT2.4*, *NRT2.5*, and *NAR2.1* in the *cipk1* mutant under nitrate deficiency, respectively (*n* = 24). Error bars indicate means ± S.E. Asterisks denote statistically significant differences according to the *t*-test (* *p* < 0.05).

Considering the low biomass and inhibited root length of the *cipk1* mutant, we speculated that *CIPK1* may affect transcriptional reprogramming and nitrate acquisition by roots under N deficiency. Whereas *NRT1.1*, *NRT2.1*, *NRT2.2*, *NRT2.4*, *NRT2.5*, and *NAR2.1* are markers monitoring N-deficiency stress response, we analyzed the expression level of these genes in the *cipk1* mutant (Figure 4). Total RNA was extracted from 7-day-old seedings germinated from 1/2 MS medium and then subjected to N deficiency treatment for 2 days. N deficiency stress repressed the expression of *NRT1.1* and induced the expression of *NRT2.1*, *NRT2.2*, *NRT2.4*, *NRT2.5*, and *NAR2.1* in WT seedlings. Strikingly, we found that the expression of *NRT1.1* and *NRT2.1* of the *cipk1* mutant was significantly lower than WT under N deficiency, while no difference was observed under N sufficient conditions. The expression of *NRT2.2*, *NRT2.4*, *NRT2.5*, and *NAR2.1* was significantly induced by N deficiency in both the *cipk1* mutant and WT, and no difference was observed between them. Taken together, our results suggested that the nitrate response and uptake system in the *cipk1* mutant may be impaired.

### 3.4. CIPK1 Interacts with TCP20 Transcription Factor

**Figure 5:**
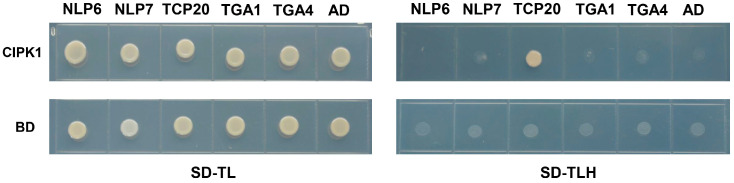
CIPK1 interacts with TCP20. Yeast two-hybrid system assays of CIPK1 with NLP6, NLP7, TCP20, TGA1, and TGA4. AD, empty bait vector; BD, empty prey vector; SD/–TL, SD medium lacking threonine and leucine; SD/–TLH, SD medium lacking threonine, leucine, and histidine.

Considering that CIPKs are functionally diverse protein kinases in plant and appear to be involved in regulating nitrate signaling and acquisition, we hypothesized that CIPK1 might interact directly with nitrate transporters or nitrate signaling transcription factors. To test this hypothesis, we employed a yeast two-hybrid assay to screen potential interacting partners of CIPK1, including NRT1.1, NRT2.1, NRT2.2, NRT2.4, NRT2.5, TCP20, NLP6, NLP7, TGA1, and TGA4. Notably, only the yeast cells co-expressing CIPK1 and TCP20 grew well on synthetic dropout medium lacking threonine, leucine, and histidine, while other experimental groups did not grow under these conditions (Figure 5). This finding suggests that CIPK1 physically interacts with TCP20.

## 4. Discussion 

Plants are sessile organisms that face dramatic fluctuations of environmental mineral nutrient availability. Therefore, they have developed sophisticated nutrient sensing systems, which activate physiological and developmental responses that prevent nutrient deficiency or toxicity. N is a fundamental macronutrient essential for higher plant growth and development. Nitrate is the primary nitrogen source for most terrestrial plants. As well as as being an essential nutrient, nitrate acts as signaling molecule to regulate the expression of hundreds of nitrate-responsive genes. In recent decades, nitrate-regulated root growth, which involves lateral root initiation, lateral root elongation, root hair growth, and primary root growth, has been well characterized [11]. When plants are exposed to mild N deficiency, the extension of primary and lateral roots is stimulated [45,46]. This systemic foraging strategy is a N-dependent root architectural adjustment to increase soil volume explored by root system.

The influence of Ca^2+^ on nitrate signaling was first reported in maize and barley. The Ca^2+^ chelator EGTA or the Ca^2+^ channel blocker La^3+^ can damage the accumulation of marker mRNAs in response to nitrate treatment [47,48]. The involvement of Ca^2+^ in nitrate signaling and acquisition has been well described in *Arabidopsis* in recent decades. Nitrate treatment can induce an increase in cytoplasmic Ca^2+^ levels, and the expression of nitrate-related genes is impaired by pretreatments with Ca^2+^ channel blockers [49]. The CBL1/9-CIPK23 complex specifically mediates high-affinity response by phosphorylating NRT1.1 at the Thr101 site under nitrate deficiency, while CIPK8 specifically engages in low-affinity responses acting as a positive regulator [26,39]. CBL7 has been found to act as a regulator in response to low nitrate conditions by manipulating the expression of *NRT2.4* and *NRT2.5* in *Arabidopsis* [40]. A novel study revealed how the nitrate signal transduction can be transmitted in a dependent Ca^2+^-pathway. Nitrate induces both Ca^2+^ accumulation in the nucleus and the rapid nuclear translocation of three CALCIUM-SENSOR PROTEIN KINASES (CPK10/30/32). Subsequently, these CPKs phosphorylate NLP7 at the Ser 205 site to affect PNR [50]. These findings suggest that Ca^2+^ multidimensionally manipulates nitrate transport, sensing, and signaling in *Arabidopsis*. Therefore, it is tempting to discover functional CBL-CIPKs involved in nitrate deficiency response.

To investigate new CBL-CIPK modules engaged in nitrate deficiency response in plants, we performed a phenotypic screening study using mutants of *cipks* and *cbls* by a reverse genetic strategy. Strikingly, we found that the *cipk1* mutant is more sensitive to nitrate starvation compared to WT, with stagnant primary roots and decreased fresh weight (Figure 1, Figure 2 and Figure 3a). To verify if the vulnerable phenotype of the *cipk1* mutant was caused by decreased N acquisition, we tested the contents of total N in WT and the *cipk1* mutant under control or nitrate deficiency conditions. We observed that the total N content was significantly decreased in the *cipk1* mutant (Figure 3b). We then analyzed the total N content of shoot and root tissues and found that it also declined in both tissues. These data showed that *CIPK1* mutation resulted in reduced N accumulation in plants (Figure 3c,d). Furthermore, the sensitive phenotype of the *cipk1* mutant was completely rescued in the transgenic complementation lines under N deficiency (Figure 1 and Figure 2). Recently, CIPK1 was found to activate and phosphorylate NAC075, leading to adapted root architecture under low nitrate availability. However, no significant difference was observed in primary root growth between WT and the *cipk1* mutants, likely due to diverse plant growth conditions [10]. In accord with our results, the overexpression of *CIPK1* led to decreased sensitivity to low nitrate in terms of root elongation. These results suggested that the loss of the *CIPK1* mutation affected the growth inhibition phenotype under nitrate deficiency.

We then analyzed the *CIPK1* mRNA level both in the shoots and roots in WT under nitrate deficiency. *CIPK1* was found to be constitutively expressed and not induced in response to nitrate deficiency. NRT1.1 regulates not only PNR but also the root foraging process, and fluctuant nitrate concentrations are sensed through NRT1.1 [51,52]. Interestingly, we found that *NRT1.1* expression was distinctly decreased in the *cipk1* mutant compared to WT under nitrate deficiency. NRT2s transporters and NAR2.1 are responsible for nitrate uptake [53]. In agreement with root phenotypes, *NRT2.1*, acting as the key regulator in root development under nitrate starvation conditions, also decreased in the *cipk1* mutant compared to WT under nitrate deficiency. To verify the machinery through which CIPK1 functions, we conducted a yeast two-hybrid assay to screen potential candidates involved in nitrate signaling and uptake processes, NRT1.1, NRT2.1, NRT2.2, NRT2.4, NRT2.5, NAR2.1, NLP6/7, TGA1/4, and TCP20, for instance. The results suggested that CIPK1 interacts with the TCP20 transcription factor (Figure 5). TCP20 interacts with NLP6/7 and directly binds to the promoters of *NRT1.1* and *NRT2.1*, integrating cell cycle-related processes and root growth. The *tcp20* mutants are defective in nitrate foraging by roots [22,54]. According to the results in our present study, CIPK1 may function in nitrate deficiency response by interacting with TCP20, although the molecular mechanism still needs to be investigated.

## 5. Conclusions

In this study, we screened *Arabidopsis thaliana* T-DNA insertion mutants of the CIPK family under nitrate defective conditions by a reverse genetic strategy. We found that the *cipk1* mutant exhibited a shorter primary root and a lower fresh weight and total N content compared with WT plants under nitrate deficiency. The *CIPK1* complementation lines completely rescued the sensitive phenotype. Additionally, the *CIPK1* mutation caused nitrogen-starvation marker genes to be decreased under nitrate deficiency. We further found that CIPK1 interacted with TCP20 in a yeast two-hybrid system. Collectively, our results reveal a novel role of CIPK1 faced with nitrate deficiency in *Arabidopsis*.

In conclusion, we provide evidence that mutations in *CIPK1* lead to significant root growth inhibition under conditions of nitrate deficiency. These findings suggest that CIPK1 may act as a new player in regulating nitrate deficiency response in plants. However, the underlying molecular mechanism of how CIPK1 cooperates with TCP20 in manipulating nitrate deficiency signaling remains to be clarified. Further research will provide insight into CIPK1′s molecular mechanism in response to nitrate deficiency, providing crop breeders with more novel insights into ways to improve NUE. 

## Data Availability

All relevant data are available from the corresponding author upon reasonable request.

## Supplementary Materials

The following supporting information can be downloaded at: https://www.mdpi.com/article/10.3390/genes15091235/s1, Figure S1: Phenotypic analysis of the *cipks* mutant germinated on LN medium; Figure S2: Expression of *CIPK1* in response to nitrate deficiency. Table S1: The primers used in gene expression analysis.
