# Supplementary material for: The Ca2+-Regulated Protein Kinase CIPK1 Modulates Plant Response to Nitrate Deficiency in Arabidopsis
Source: Genes (Basel). 2024 Sep 23;15(9):1235. doi: 10.3390/genes15091235 (PMC11431708; doi:10.3390/genes15091235)
Supplement: Supplementary file 1 [file genes-15-01235-s001.zip › genes-3208561-supplementary.pdf]

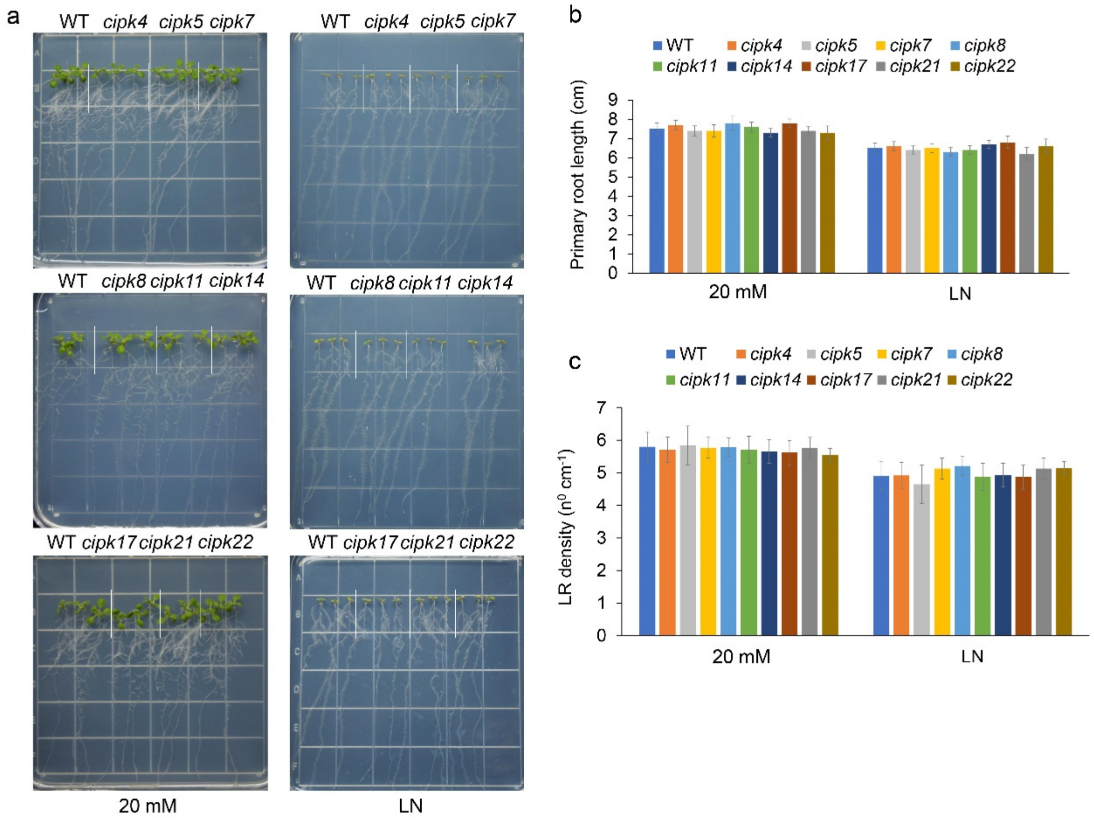

Figure S1. Phenotypic analysis of the *cipks* mutant germinated on LN medium. (a) Phenotypic analysis of the *cipk4*, *cipk5*, *cipk7*, *cipk8*, *cipk11*, *cipk14*, *cipk17*, *cipk21* and *cipk22* mutants germinated from 0, 0.05, 0.1, 1, and 20 mM NO_3^-. (b) Primary root length of the tested lines under the different nitrate treatments (n = 24). (c) Lateral root density under the different nitrate treatments (n = 24). Error bars represent means ± S.E. Asterisks indicate statistically significant differences according to t-test.

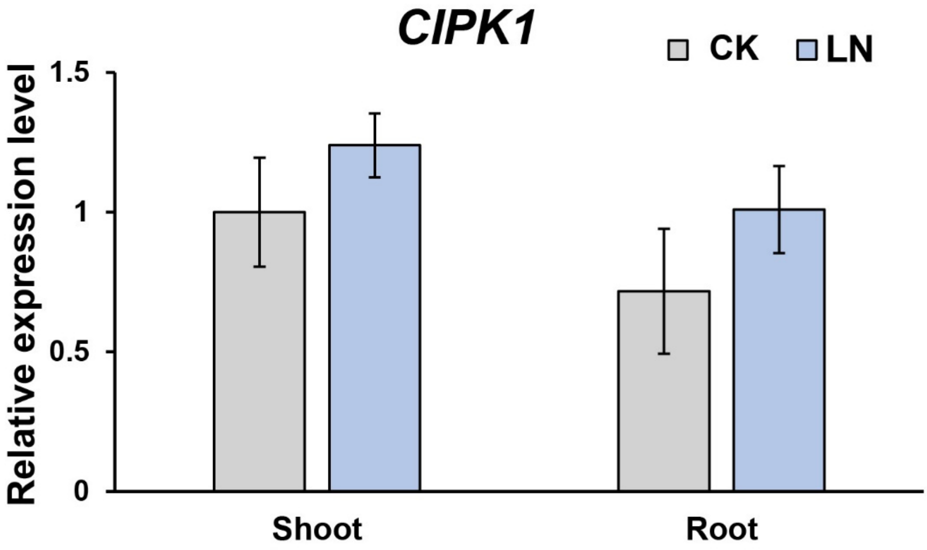

Figure S2. Expression of *CIPK1* in response to nitrate deficiency. Expression of *CIPK1* in shoot and root are analysed under nitrate deficiency. (n = 24). Error bars represent means ± S.E. Asterisks indicate statistically significant differences according to t-test.

Table S1. Primers used in RT-PCR

Primer	Sequence（5′-3′）
Actin-F	TTCAGCTTCATCGGCCGTTGCATTT
Actin-R	CATCAGCATCGGCCATGGTCTACGA
NRT1.1-F	TCATCGCCGATACCTTTCTC
NRT1.1-R	CGGAATTGTTCAGTGTGTGG
NRT2.1-F	TCCACAGATCCAGTGAAAGGTAC
NRT2.1-R	TGACTTGGCGTTCTCGGCA
NRT2.2-F	TTGCTGATGGAGCCAAGACC
NRT2.2-R	TCAATGACACATGATCAAGCATTG
NRT2.4-F	GCTTGTGGAGCTACCTTTGC
NRT2.4-R	GAAGTTTCCACCAGCTCCAG
NRT2.5-F	AGGATCGACATACACGAGAGAGACG
NRT2.5-R	GGCTTCCGATATGTAAGTTCTTTTC
NAR2.1-F	GACCTGCCCACACAAGATCA
NAR2.1-R	TGGAGGCAATATCTAGGGACGC
